# Longitudinal association between daytime sleepiness and cognitive decline in dementia: A study protocol

**DOI:** 10.1192/j.eurpsy.2021.1364

**Published:** 2021-08-13

**Authors:** J. Silva, A.R. Ferreira, L. Fernandes

**Affiliations:** 1 Fmup, Faculty of Medicine, University of Porto, Porto, Portugal; 2 Cintesis - Center For Health Technology And Services Research, Faculty of Medicine, Porto University, Porto, Portugal; 3 Cintesis – Center For Health Technology And Services Research; Department Of Clinical Neuroscience And Mental Health, Faculty of Medicine of Porto University; Centro Hospitalar Universitário de São João, Porto, Portugal, Portugal

**Keywords:** dementia, Daytime sleepiness, Cognitive decline, longitudinal study

## Abstract

**Introduction:**

Dementia is a major cause of disability worldwide. About 25%-40% of patients with mild to moderate dementia are affected by sleep-awake cycle disturbances, including increased daytime sleepiness and insomnia. However, little is known about the specific impact of excessive daytime sleepiness on the cognitive decline of dementia patients.

**Objectives:**

To evaluate the impact of daytime sleepiness on the cognitive decline of dementia patients. Additionally, longitudinal associations with functional impairment and neuropsychiatric symptoms will be explored.

**Methods:**

A longitudinal study will be conducted in a psychogeriatric consultation. Patients will be consecutively invited according to predefined eligibility criteria. Those aged ≥65 years, with dementia diagnosis or Mini-Mental State Examination (MMSE) <24, and with a knowledgeable caregiver, will be included. The exclusion criteria are: a caregiver <18 years, terminally ill, incapable to communicate or with a known diagnosis of insomnia, sleep related respiratory disorders, central hyperinsomnia, restless legs syndrome or sleep paralysis. Participants will undergo an assessment with a comprehensive protocol including: Montreal Cognitive Assessment (MoCA), Barthel and Lawton Index, Epworth Sleepiness Scale (ESS), Neuropsychiatric Inventory (NPI) and Global Deterioration Scale (GDS). Participants will be re-assessed 6 months after the initial evaluation. The Health Ethics Committee of Hospital Universitário de São João granted the study authorization (nº 260/2020).

**Results:**

Findings will be disseminated via publication in peer-reviewed journals and presentations at national and international scientific conferences.

**Conclusions:**

This study will address key questions on the relation of daytime sleepiness and dementia outcomes, in order to undertake corrective and preventive non-pharmacological and pharmacological approaches.

